# A New Prognostic Instrument for Predicting the Probability of Completion of Cisplatin during Chemoradiation for Head and Neck Cancer

**DOI:** 10.3390/jpm13071120

**Published:** 2023-07-10

**Authors:** Dirk Rades, Inga Zwaan, Christian Idel, Ralph Pries, Karl L. Bruchhage, Samer G. Hakim, Nathan Y. Yu, Tamer Soror

**Affiliations:** 1 Department of Radiation Oncology, University of Lubeck, 23562 Lubeck, Germany; inga.zwaan@uksh.de (I.Z.); tamer.soror@uksh.de (T.S.); 2 Department of Oto-Rhino-Laryngology & Head and Neck Surgery, University of Lubeck, 23562 Lubeck, Germany; christian.idel@uksh.de (C.I.); ralph.pries@uksh.de (R.P.); karl-ludwig.bruchhage@uksh.de (K.L.B.); 3 Department of Oral and Maxillofacial Surgery, University of Lubeck, 23562 Lubeck, Germany; 4 Department of Oral and Maxillofacial Surgery, MSH Medical School Hamburg, Schwerin Campus, 19055 Schwerin, Germany; 5 Department of Radiation Oncology, Mayo Clinic, Phoenix, AZ 85054, USA; yu.nathan@mayo.edu

**Keywords:** head and neck cancer, chemoradiation, cisplatin, completion of treatment, prognostic instrument

## Abstract

Many head and neck cancer patients assigned to definitive or adjuvant chemoradiation treatment do not complete the concurrent cisplatin dose. We determined corresponding risk factors and developed a prognostic instrument to help identify these patients. Ten pre-treatment characteristics were retrospectively analyzed in 154 patients with head and neck cancer who were treated via chemoradiation with cisplatin. These pre-treatment characteristics included age, sex, Karnofsky performance score, tumor site, primary tumor stage, nodal stage, histologic grade, upfront surgery, human papilloma virus status, and history of smoking. The characteristics significantly associated with the completion of cisplatin-based treatment, the receipt of ≥80% cisplatin, or showing a strong trend of association after multivariate analyses were used for the prognostic instrument. For each characteristic, 0 points were assigned for worse outcomes, and 1 point was assigned for better outcomes. Patients’ scores were calculated by adding these points. Age ≤ 60 years and a Karnofsky performance score of 90–100 were significantly associated with both endpoints after multivariate analysis, and male gender showed a trend for association with the receipt of ≥80% cisplatin. Patient scores were 0, 1, 2, and 3 points. The corresponding rates of completion of cisplatin-based treatment were 14%, 41%, 62%, and 72%, respectively (*p* = 0.004). The rates of receipt of ≥80% cisplatin were 29%, 54%, 72%, and 94%, respectively (*p* < 0.001). This new prognostic instrument helps to predict whether head and neck cancer patients scheduled for chemoradiation will receive cisplatin as planned.

## 1. Introduction

Many patients with locally advanced squamous cell carcinoma of the head and neck (SCCHN) receive cisplatin-based chemoradiation as a form of adjuvant or definitive treatment [1,2,3,4]. The most common concurrent regimen of concurrent chemotherapy includes 100 mg/m^2^ of cisplatin every 3 weeks [1,2,3]. A considerable proportion of these patients are not able to receive the complete chemotherapy treatment as planned; acute toxicity may even lead to the discontinuation of radiotherapy [5]. Both situations have a negative impact on disease outcomes [4,5,6]. Therefore, several attempts have been made to reduce treatment-related toxicity, including the use of alternative chemotherapy regimes with or without cisplatin [7,8,9,10,11]. A retrospective study from 2016 compared a dose of 100 mg/m^2^ of cisplatin on day 1 every 3 weeks to weekly cisplatin with doses of 30–40 of mg/m^2^ for the chemoradiation of locally advanced SCCHN and found that cisplatin given every 3 weeks was associated with significantly better loco-regional control and overall survival, albeit with increased acute toxicity [8]. In a phase III non-inferiority trial from 2018, 100 mg/m^2^ of cisplatin every 3 weeks was superior to a weekly 30 mg/m^2^ dose of cisplatin in terms of loco-regional control; however, increased toxicity was observed again [9]. In 2016, a retrospective study compared two courses of 20 mg/m^2^ cisplatin on days 1–5 and three courses of 100 mg/m^2^ on day 1 in 230 patients with locally advanced head and neck cancer [10]. The three-course regimen was not superior regarding loco-regional control, metastases-free survival, and overall survival but was associated with significantly increased rates of pneumonia/sepsis, nausea/vomiting, and nephrotoxicity. Therefore, the two-course regimen (20 mg/m^2^ on days 1–5) was considered preferable. Recently, two courses of 20 mg/m^2^ cisplatin on days 1–5 was compared with two courses of 25 mg/m^2^ on days 1–4 [4]. Both regimens were associated with similar acute toxicity, loco-regional control, and overall survival; therefore, these regimens were used for our present study. 

Receipt of <80% of the planned cisplatin dose is associated with significantly worse overall survival [4]. Receipt of planned cisplatin during concurrent chemoradiation for head and neck cancer has a clear association with treatment outcomes [11,12,13,14,15,16,17,18]. It is important to identify patients who will not be able to receive the complete planned dose of cisplatin prior to the start of chemoradiation. These patients may require closer monitoring, more intensive supportive care, or radiotherapy alone with alternative dose fractionation regimens [19,20,21,22,23]. The present study aimed to determine the risk factors associated with the non-completion of cisplatin-based chemotherapy and develop a corresponding risk score in patients assigned to concurrent chemoradiation treatment for head and neck cancer. 

## 2. Materials and Methods

The data of 154 patients with locally advanced head and neck cancer who had appropriate renal function for treatment with cisplatin and were scheduled for cisplatin-based concurrent chemoradiation between 2012 and 2022 were retrospectively evaluated in terms of whether they completed the planned chemotherapy. The study received approval from the Ethics Committee of the University of Lübeck (file number 21-034). A total of 144 patients were scheduled for 60–70 Gy of external beam radiotherapy (EBRT). Dose regimens of EBRT included 60 Gy following microscopically complete resection, 66 Gy following microscopically incomplete resection or in the case of an extracapsular extension of lymph node metastases, and 70 Gy for definitive treatment or following macroscopically incomplete resection. In all patients undergoing upfront surgery, at least microscopically complete resection was achieved. Three patients could not receive the radiation dose as planned. Among these patients, the total doses were 8 Gy, 16 Gy, and 22 Gy, respectively. The median dose of EBRT in the entire cohort was 66 Gy. Twelve patients received a brachytherapy boost to the region of the primary tumor in addition to 50–66 Gy of conventionally fractionated EBRT given to primary tumor and lymph nodes. The brachytherapy boost included two fractions of 2.5 or 3.0 Gy per day and a total of three to five fractions. Of the twelve patients receiving a brachytherapy boost, nine patients had cancer of the oropharynx, and three patients had cancer of the oral cavity. Indications for a brachytherapy boost included close or positive margins following resection, lymphatic invasion, tumor infiltration > 5 mm, and individualized treatment for tumors of the lateral tongue or the base of the tongue. Radiotherapy was planned to be run in conjunction with two cycles of concurrent chemotherapy with 20 mg/m^2^ cisplatin on days 1–5 in 52 patients or 25 mg/m^2^ cisplatin on days 1–4 in 102 patients. These regimens represented the standard approach in our institution [4,10]. A total of 81 patients were treated via adjuvant chemoradiation following a surgical procedure that included the resection of a primary tumor and neck dissection; 73 patients received definitive chemoradiation. 

Ten pre-treatment characteristics were analyzed in addition to the EBRT dose, the radiation technique (EBRT alone vs. EBRT plus brachytherapy), and the cisplatin regimen (20 mg/m^2^ on days 1–5 vs. 25 mg/m^2^ on days 1–4). These pre-treatment characteristics included the following: age at the time of EBRT (≤60 years vs. ≥61 years), sex (female vs. male), Karnofsky performance score (50–80 vs. 90–100), main tumor site (oropharynx vs. oral cavity vs. hypopharynx vs. larynx vs. more than 1 site), primary tumor stage (T1–3 vs. T4), nodal stage (N0–1 vs. N2–3), histologic grade (G1–2 vs. G3), upfront surgery (no vs. yes), human papilloma virus (HPV) status (negative vs. positive), and history of smoking (no vs. yes). Regarding the categorization of main tumor sites, patients were classified as “more than 1 site” if the main tumor site could not be clearly identified. This classification was used for a total of 26 patients (Table 1). Ten of these patients had cancer of the oropharynx and hypopharynx, eight patients had cancer of the hypopharynx and larynx, four patients had cancer of the oropharynx and oral cavity, and four patients had cancer of the oropharynx, hypopharynx, and larynx. Boost doses were equally administered to all sites affected by the primary tumor. 

Investigated endpoints included the completion of planned chemotherapy and receipt of ≥80% of the planned cumulative cisplatin dose. The pre-treatment characteristics that were significantly associated with at least one endpoint or showed a trend for association following multivariate analyses were used to develop a prognostic instrument to estimate the probability of completing cisplatin-based chemotherapy. Each characteristic was measured on a points scale of “0” (worse outcomes) or “1” (better outcomes). To obtain the individual patient scores, the points of the significant characteristics were added. Based on the individual patient scores, prognostic groups were designed. Statistical analyses were performed using Pearson’s chi-squared test or, if the number of patients was <5 in at least one cell, Fisher’s exact test. A multivariable logistic regression was used to evaluate the prognostic effect of the baseline characteristics simultaneously; the Wald chi-squared test was used to judge their statistical significance. 

In addition, the prognostic groups were evaluated in terms of loco-regional control and overall survival. These analyses were performed using the Kaplan–Meier method and the log-rank test. For all statistical analyses described above, *p*-values < 0.05 were considered statistically significant, and *p*-values ≤ 0.06 indicated a trend. 

## 3. Results

In the entire cohort, 68 patients (44%) did not complete the planned chemotherapy regime, and 47 patients (31%) received less than 80% of the planned cisplatin dose. The main reasons for this included decreased renal function, leukopenia, and infections. In our univariable analyses, the completion of the planned chemotherapy regime (Table 1) was significantly associated with a Karnofsky performance score of 90–100 (*p* = 0.021) and upfront surgery (*p* = 0.012). In addition, trends were observed for age ≤ 60 years (*p* = 0.060) and male gender (*p* = 0.052). In the subsequent multivariable analysis, age ≤ 60 years (*p* = 0.036) and a Karnofsky performance score of 90–100 (*p* = 0.010) achieved significance, whereas male gender (*p* = 0.10) and upfront surgery (*p* = 0.98) were not significant (Table 1). 

In our univariable analyses, receipt of at least 80% of the planned cumulative cisplatin dose (Table 2) was significantly associated with a Karnofsky performance score of 90–100 (*p* = 0.007), upfront surgery (*p* = 0.019), and age ≤ 60 years (*p* = 0.002). In the subsequent multivariable analysis, age ≤ 60 years (*p* = 0.011) and a Karnofsky performance score of 90–100 (*p* = 0.004) achieved significance, and male gender (*p* = 0.052) showed a trend. Upfront surgery (*p* = 0.60) was not significant in the multivariable analysis (Table 2). 

Since age, Karnofsky performance score, and gender were significantly associated or showed a trend for an association with one or both investigated endpoints, these three characteristics were used for the prognostic instrument. 

One point was assigned to age ≤ 60 years, a Karnofsky performance score of 90–100, and male gender, respectively. Zero points were given for age ≥ 61 years, a Karnofsky performance score of ≤80, and female gender, respectively. After totaling the corresponding points, the patient scores were 0 points (*n* = 7), 1 point (*n* = 46), 2 points (*n* = 69), or 3 points (*n* = 32). Each score represented a prognostic group. For these patient scores, the rates of completion regarding cisplatin-based treatment were 14% (0 points), 41% (1 point), 62% (2 points), and 72% (3 points), respectively (*p* = 0.004), and the rates of receipt of ≥80% cisplatin were 29%, 54%, 72%, and 94%, respectively (*p* < 0.001) (Table 3). Thus, the accuracy (positive predictive values) values of the prognostic instrument with respect to predicting completion of cisplatin-based treatment and receipt of ≥80% of the planned cisplatin dose were 72% and 94%, respectively. The accuracy values for predictions regarding the non-completion of cisplatin-based treatment and the receipt of only <80% of the planned cisplatin dose were 86% and 71%, respectively. 

The median follow-up times were 28.5 months (range: 0–112 months in the entire cohort and 33 months—range: 6–112 months—in those patients who were alive at the time of the last contact). According to the additional analyses of the four prognostic groups with respect to treatment outcomes, no significant differences were found for loco-regional control (*p* = 0.63) and overall survival (*p* = 0.29). The one-year and two-year loco-regional control rates were 86% and 86% (0 points), 78% and 71% (1 point), 86% and 82% (2 points), and 81% and 76% (3 points), respectively. And the one-year and two-year overall survival rates were 86% and 86% (0 points), 85% and 73% (1 point), 88% and 85% (2 points), and 94% and 86% (3 points), respectively. The points for loco-regional control and overall survival are summarized in Table 3.

## 4. Discussion

Many patients with head and neck cancer are scheduled for treatment consisting of concurrent chemoradiation with cisplatin, which is often associated with significant acute toxicities [5]. These toxicities may be so severe to an extent where the radiotherapy treatment needs to be interrupted or even completely stopped. In terms of loco-regional control and overall survival, this was shown to compromise treatment outcomes [4,6]. If chemoradiation with cisplatin is not possible, other single agents or combinations may be used [7,24,25,26]. If the patients will likely not be able to receive the complete concurrent treatment, even if cisplatin is replaced by another systemic therapy, radiotherapy alone with an alternative dose fractionation could be an option [19,20,21,22,23,27]. These alternative regimens have shorter overall treatment times compared to conventional fractionation (2.0 Gy per fraction on five consecutive days per week). The negative impact of longer overall treatment times on the outcomes of radiotherapy of head and neck cancers was shown about 35 years ago [28]. Moreover, in a retrospective study of 80 patients receiving definitive treatment of locally advanced head and neck cancer, accelerated fractionation with a concomitant boost (69.6 Gy in 39 fractions over 5.5 weeks) was not inferior to conventional cisplatin-based chemoradiation (70.0 Gy in 35 fractions over 7 weeks) with respect to loco-regional control, metastases-free survival, and overall survival, but was associated with fewer interruptions of radiotherapy [20]. Moreover, 38.9% of the patients in the chemoradiation group could not complete planned chemotherapy course. To provide the optimal treatment for head and neck cancer patients being assigned to chemoradiation, it is important to know, as precisely as possible, whether a patient will be able to receive the complete concurrent cisplatin dose (cumulative dose 200 mg/m^2^) or at least 80% of the planned dose [4,11,14,15,16,17,18].

Therefore, we aimed to identify prognostic factors regarding the completion of concurrent cisplatin and receipt of ≥80% of the planned cisplatin dose. Another goal of this study was to develop a prognostic instrument based on these factors. According to our results, the completion of planned cisplatin-based treatment was independently associated with age ≤ 60 years and a Karnofsky performance score of 90–100. Moreover, receiving ≥80% of the planned cisplatin dose was independently associated with age ≤ 60 years and a Karnofsky performance score of 90–100, and male gender showed a strong trend. These findings are in line with the results of the very few previous studies that have investigated the completion rates of chemoradiation, radiotherapy, or chemotherapy in patients treated for a malignant disease. In a retrospective study by Nakano et al. in which 159 patients receiving concurrent cisplatin-based chemoradiation for head and neck cancer were included, age ≥ 61 years was not significantly associated (*p* = 0.15) with decreased tolerance to treatment [29]. Wang et al. retrospectively analyzed the data of 487 patients receiving treatment via definitive chemoradiation for locally advanced esophageal cancer [30]. In this study, older age was an independent predictor of non-completion of concurrent chemotherapy. In another retrospective study, advanced age was negatively associated with the completion of standard radiotherapy in patients with glioblastoma [31]. Moreover, older age was significantly associated with discontinuation of radiotherapy in a retrospective study of patients irradiated for lung cancer [32]. In that study, discontinuation of radiotherapy was also associated with decreased pulmonary function, which is generally associated with a worse performance score due to shortness of breath. Moreover, in a retrospective study of women who were treated with adjuvant intraperitoneal and intravenous chemotherapy following cytoreductive surgery, non-completion of chemotherapy was significantly associated with worse Eastern Cooperative Oncology Group performance score (*p* = 0.04) [33]. In the above-mentioned retrospective study by Nakano et al. that included 159 patients, significantly more male than female patients tolerated higher doses of cisplatin (odds ratio = 25.00, *p* = 0.005) [29]. 

In the present study, the three characteristics that were significantly associated with the completion of planned cisplatin-based chemotherapy and/or receipt of ≥80% of the planned cisplatin dose were used to create the prognostic instrument. The resulting scoring points ranged between 0 and 3. A lower score represented a lower probability of completing the planned chemotherapy or receiving ≥80% of the planned dose. The corresponding probabilities of patients with 0 points were only 14% and 29%, respectively. Therefore, the assignment of cisplatin-based concurrent chemoradiation needs to be carefully reviewed in these patients. If the addition of cisplatin to radiotherapy leads to increased acute toxicity and consequently disrupts radiation treatment for more than one week, the treatment outcomes become significantly worse [4,6]. Thus, patients with 0 points according to our new prognostic instrument may benefit from combining radiotherapy with systemic agents other than cisplatin or from radiotherapy alone with alternative dose fractionation regimens associated with a shortened overall treatment time [19,20,21,22,23,27]. This may also apply to some patients belonging to the 1-point group, since the probabilities of cisplatin-based chemotherapy completion and receipt of ≥80% of the planned dose in this group were 41% and 54%, respectively, i.e., not optimal. In contrast, patients in the 2-points group (with probabilities of 62% and 72%, respectively) and particularly patients of the 3-points group (with probabilities of 72% and 94%, respectively) have a greater chance of completing their planned treatment and therefore appear good candidates for cisplatin-based chemoradiation. However, the higher rates of completing the planned chemotherapy or receiving ≥80% of the planned dose were not associated with significantly improved outcomes in terms of loco-regional control and overall survival. To a certain extent, this may be explained by the fact that a considerable proportion (28%) of the patients achieving the highest score of 3 points did not receive the complete cisplatin dose. Moreover, considering the cisplatin regimens used in the present study, receipt of ≥80% of the planned cisplatin dose means a cumulative dose of ≥160 mg/m^2^. Even though a previous study suggested that cumulative doses ≥ 160 mg/m^2^ were associated with improved 3-year LRC and 3-year progression-free survival when compared to <160 mg/m^2^ in patients with nasopharynx cancer and detectable Epstein–Barr virus (EBV) DNA following the induction of chemotherapy [16], other studies and a systematic review by Strojan et al. have shown that the cumulative dose should be at least 200 mg/m^2^ [11,14,15,17]. In a study by Oliva et al., which investigated patients with HPV-positive oropharynx cancer, cumulative cisplatin doses of <200 mg/m^2^ were associated with worse cancer-specific survival than doses ≥ 200 mg/m^2^ in the subgroup of patients with stage III disease (*p* = 0.02) [15]. In a study including patients with nasopharynx cancer by Gundog et al., a cumulative cisplatin dose of ≥200 mg/m^2^ was an independent predictor of improved overall survival (*p* = 0.03) [14]. In a study by Babar et al., cumulative cisplatin dose of ≥200 mg/m^2^ resulted in significantly better disease-free survival (*p* = 0.007) in patients receiving chemoradiation following the resection of high-risk oral cavity cancer [17]. After considering the results of these studies, the factor of receipt of ≥80% of the planned cisplatin dose used in the present study (≥160 mg/m^2^) may not be sufficient to achieve improvements in loco-regional control and overall survival. Thus, it may be reasonable to evaluate our new scoring system in a cohort of patients with planned cumulative cisplatin doses > 200 mg/m^2^. Moreover, a larger patient cohort and a longer period of follow-up are required to properly answer the question of whether a greater chance of completing planned chemotherapy is associated with better prognoses. Moreover, when considering these suggestions and recommendations, one should keep in mind that the prognostic instrument was created from retrospective data. Retrospective studies always bear the risk of hidden selection biases. Thus, the instrument needs to be validated in a prospective cohort and, until then, can mainly serve for coarse orientation when aiming to identify patients with head and neck cancer who may not be able to tolerate cisplatin-based chemoradiation sufficiently well.

## 5. Conclusions

A new prognostic instrument was created that helps to predict whether head and neck cancer patients who are scheduled for chemoradiation will be able to complete their cisplatin-based chemotherapy course as planned or at least 80% of the planned dose. These predictions may contribute to optimal treatment personalization. Since the instrument was developed from retrospective data, it should be validated in a larger prospective patient cohort. Moreover, a longer period of follow-up is required to properly identify the potential benefits of completing chemotherapy in terms of improved treatment outcomes.

## Figures and Tables

**Table 1:** Associations between patient characteristics and completion of cisplatin-based chemotherapy as planned.

Characteristic	Completion of Chemotherapy with Cisplatin as Planned		Univariable *p*-Value	Multivariable *p*-Value
	No, *n* (%)	Yes, *n* (%)		
Age			0.060	0.035
≤60 years	26 (38)	46 (53)		
≥61 years	42 (62)	40 (47)		
Sex			0.052	0.11
Female	18 (26)	12 (14)		
Male	50 (74)	74 (86)		
Karnofsky performance status			0.021	0.009
50–80	38 (56)	32 (37)		
90–100	30 (44)	54 (63)		
Main tumor site			0.12	0.57
Oropharynx	33 (49)	41 (48)		
Oral cavity	5 (7)	17 (20)		
Hypopharynx	6 (9)	7 (8)		
Larynx	8 (12)	11 (13)		
>1 site	16 (24)	10 (12)		
Primary tumor stage			0.59	0.44
T1–3	35 (51)	48 (56)		
T4	33 (49)	38 (44)		
Nodal stage			0.61	0.73
N0–1	18 (26)	26 (30)		
N2–3	50 (74)	60 (70)		
Histologic grade ^a^			0.75	0.37
G1–2	39 (58)	45 (56)		
G3	28 (42)	36 (44)		
Upfront surgery			0.012	0.94
No	40 (59)	33 (38)		
Yes	28 (41)	53 (62)		
HPV status ^b^			0.71	0.71
Negative	30 (61)	34 (58)		
Positive	19 (39)	25 (42)		
History of smoking ^c^			0.94	0.57
No	9 (13)	11 (13)		
Yes	59 (87)	75 (87)		
Brachytherapy			0.67	0.47
No	62 (91)	80 (93)		
Yes	6 (9)	6 (7)		
Dose of EBRT			0.067	0.73
<68 Gy	36 (53)	58 (67)		
68–70 Gy	32 (47)	28 (33)		
Cisplatin regimen			0.17	0.51
20 mg/m^2^/d1–5	27 (40)	25 (29)		
25 mg/m^2^/d1–4	41 (60)	61 (71)		

EBRT: external beam radiotherapy; HPV: human papilloma virus; ^a^ unknown in 6 patients; ^b^ unknown in 46 patients; ^c^ unknown in 16 patients.

**Table 2:** Associations between patient characteristics and administration of ≥80% of the planned cisplatin dose.

Characteristic	Administration of ≥80% of the Planned Cisplatin Dose		Univariate *p*-Value	Multivariable *p*-Value
	No, *n* (%)	Yes, *n* (%)		
Age			0.002	0.012
≤60 years	13 (28)	59 (55)		
≥61 years	34 (72)	48 (45)		
Sex			0.089	0.052
Female	13 (28)	17 (16)		
Male	34 (72)	90 (84)		
Karnofsky performance status			0.007	0.004
50–80	29 (62)	41 (38)		
90–100	18 (38)	66 (62)		
Main tumor site			0.53	0.49
Oropharynx	20 (43)	54 (50)		
Oral cavity	5 (11)	17 (16)		
Hypopharynx	5 (11)	8 (7)		
Larynx	6 (13)	13 (12)		
>1 site	11 (23)	15 (14)		
Primary tumor stage			0.91	0.076
T1–3	25 (53)	58 (54)		
T4	22 (47)	49 (46)		
Nodal stage			0.87	0.68
N0–1	13 (28)	31 (29)		
N2–3	34 (72)	76 (71)		
Histologic grade ^a^			0.45	0.88
G1–2	24 (52)	60 (59)		
G3	22 (48)	42 (41)		
Upfront surgery			0.019	0.61
No	29 (62)	44 (41)		
Yes	18 (38)	63 (59)		
HPV status ^b^			0.30	0.81
Negative	22 (67)	42 (56)		
Positive	11 (33)	33 (44)		
History of smoking ^c^			0.27	0.77
No	4 (9)	16 (15)		
Yes	43 (91)	91 (85)		
Brachytherapy			1.00	0.23
No	43 (91)	99 (93)		
Yes	4 (9)	8 (7)		
Dose of EBRT			0.093	0.095
<68 Gy	24 (51)	70 (65)		
68–70 Gy	23 (49)	37 (35)		
Cisplatin regimen			0.43	0.97
20 mg/m^2^/d1–5	18 (38)	34 (32)		
25 mg/m^2^/d1–4	29 (62)	73 (68)		

EBRT: external beam radiotherapy; HPV: human papilloma virus; ^a^ unknown in 6 patients; ^b^ unknown in 46 patients; ^c^ unknown in 16 patients.

**Table 3:** Associations between scoring points and outcomes in terms of completion of cisplatin-based chemotherapy as planned, administration of ≥80% of the planned cisplatin dose, loco-regional control, and overall survival.

Outcome	Scoring Points				*p*-Value
	0 Points	1 Point	2 Points	3 Points	
Completion of chemotherapy with cisplatin as planned	14%	41%	62%	72%	0.004
Administration of ≥80% of the planned cisplatin dose	29%	54%	72%	94%	<0.001
1-year loco-regional control 2-year loco-regional control	86% 86%	78% 71%	86% 82%	81% 76%	0.63
1-year overall survival2-year overall survival	86% 86%	85% 73%	88% 85%	94% 86%	0.29

## Data Availability

The data pertaining to this study cannot be shared due to data protection regulations. According to the responsible ethics committee, only evaluations of anonymized data are allowed.
